# Youth and social cohesion in times of the COVID pandemic: Most negatively affected? Most resilient?

**DOI:** 10.3389/fpsyg.2023.1036516

**Published:** 2023-02-20

**Authors:** Carina Hartz, Georgi Dragolov, Regina Arant, Jan Delhey, Kai Unzicker, Klaus Boehnke

**Affiliations:** ^1^Bremen International Graduate School of Social Sciences (BIGSSS), Constructor University, Bremen, Germany; ^2^Bremen International Graduate School of Social Sciences (BIGSSS), Universität Bremen, Bremen, Germany; ^3^School of Business, Social and Decision Sciences, Constructor University, Bremen, Germany; ^4^Otto von Guericke University Magdeburg, Magdeburg, Germany; ^5^Bertelsmann Stiftung, Gütersloh, Germany

**Keywords:** youth, social cohesion, optimism, COVID, resilience

## Abstract

**Introduction:**

The current article reports findings from three large representative survey studies in the German federal state of Baden-Württemberg. The studies are part of the *Social Cohesion Radar* research initiative of Bertelsmann Stiftung.

**Methods:**

The article explores the role of social cohesion in the relationship between COVID-based objective and subjective strain, on the one hand, and future optimism for the youth, citizens of active age, and the elderly. In particular, it focuses on the question whether the degree of social cohesion perceived by respondents moderates the relationship between strain and optimism in the different age groups.

**Results and discussion:**

Findings show that the impact of perceived social cohesion in people’s life context has only modest effects on the relationship between strain and future optimism. Yet, the results show that having been affected by COVID in one way or the other leads to a small but persistent bounce-back effect. People affected by COVID tend to look more optimistic into the future than those who were not.

## Introduction

The Corona pandemic has posed a challenge to societies around the globe since its outbreak in Spring 2020. By now, nearly 600 million people worldwide (some 32 million in Germany) have been infected with the virus, of whom almost 6.5 million died.^1^ The course of the disease often varied by health status. Symptoms were barely noticeable for some, while others had to be hospitalized. Many witnessed close others contracting the virus, some of whom suffered severe symptoms or passed away. The measures of the authorities against COVID-19 required citizens to change their behavior and mindset. Social distancing disrupted the daily lives of communities (Fetzer et al., 2020). Schools and universities switched to an online teaching mode; homes had to be converted into workspaces. Many employees were put on reduced working hours or even forced to quit their jobs. Contacts with family and friends were curbed massively, repeated lockdowns made most leisure activities impossible.

Against the background of these objective strains induced by COVID, it is little surprise that research on mental health has surged during the pandemic (Holmes et al., 2020). A consistent finding of the available studies is an increase in subjective strains – a variety of adverse psychological conditions (see, e.g., the systematic review by Xiong et al., 2020). The pandemic has been found to be detrimental to mental health, raising depression, and anxiety (Kwong et al., 2020; Pierce et al., 2020; Tsamakis et al., 2020; OECD, 2021), and to be associated with social isolation, financial insecurity, and psychological distress (Robillard et al., 2020; OECD, 2021).

Undoubtedly, the combination of objective and subjective strains induced by COVID presents a stressful situation to individuals. Moreover, 3 years later by now, the future course still remains uncertain. This raises the question as to how individuals look into the future given their experiences in the Corona pandemic. Research on the topic is rather scarce. Drawing on a convenience sample of adults in Turkey, Arslan et al. (2021) show that respondents tend to react to stress during the pandemic with more inflexibility, greater pessimism, and lower optimism. Does the finding of lower optimism for the future apply to the context of Germany, too?

In the present article, we explore the relationship between COVID-affectedness and optimism for the future in Germany. In the German public discourse on COVID, the message was clear: First and foremost, citizens must protect the weakest in society, namely the elderly and chronically ill. This necessitated that everyone without exception observe the preventive measures which dramatically restricted social interaction. As a result of the strict regulations, most of the elderly seemed to have felt protected, whereas their younger counterparts tended to see their life and future plans as stalled, to say the least. These opposing views on the situation possibly indicate intergenerational differences. We, thus, investigate whether the relationship between COVID-affectedness and future optimism differs across age groups. However, although individuals were all subjected to the COVID situation, it is conceivable that not all of them were equally strongly affected by it. The relationship between COVID-affectedness and future optimism can be mitigated by various possible “protective” factors that increase individuals’ resilience against adverse situations. Therefore, we explore whether the experience of strong social cohesion—the perceived quality of social interactions in the life context of individuals (Dragolov et al., 2016)–reduces the negative impact of COVID-affectedness.

The empirical basis of our research consists of three independent representative survey studies conducted in 2017, 2019, and December 2021/January 2022 in the German federal state of Baden-Württemberg as part of the *Social Cohesion Radar* (SCR) of Bertelsmann Stiftung. The SCR is an established, theoretically grounded and methodologically sound measurement instrument for the quantitative assessment of the degree of social cohesion in a social entity as perceived by individuals along three domains (each subdivided into three dimensions). These are social relations (social networks, trust in people, acceptance of diversity), connectedness with the social entity (identification, trust in institutions, perception of fairness), and focus on the common good (solidarity and helpfulness, respect for social rules, civic participation). The tool calculates scores for each of the nine dimensions. Dimension scores are subsequently averaged to one single formative index of social cohesion that can range from zero (weakest possible degree) to 100 (strongest possible degree).

The study series on social cohesion in Baden–Württemberg began as part of an all-German study in 2017 (Arant et al., 2017). Two further surveys with larger samples of residents of the federal state followed in 2019 (Dragolov et al., 2020) and at the turn of the years 2021 and 2022 (Boehnke et al., 2022). Those fielded in 2019 and in 2021/22 were commissioned by the Ministry of Social Affairs, Health, and Integration of Baden-Württemberg. The grand sample encompasses data from a three-wave cross-sectional time series (details below). To preview one core finding already here: Whereas between 2017 and 2019 social cohesion in Baden-Württemberg was very stable, changing less than one point from 63.0 up to 63.8, drastic changes occurred from 2019—thus, before COVID—to 2022: Social cohesion dropped by almost 10 points from 63.8 down to 54.3. This suggests that COVID-19 had detrimental consequences for social cohesion in Baden-Württemberg. While no age-related differences in the overall assessment of social cohesion between young people and the rest of the population were observed, the most recent data point to an interesting slightly paradoxical finding regarding COVID: Study participants from all ages (including youth themselves) considered adolescents and emerging adults to be the age group most strongly affected by COVID. And indeed, the study results presented in this paper show substantial differences between youth and older citizens in self-reports on infection rates (17% vs. 6%, respectively) and severe symptoms (7% vs. 2%, respectively). Youth also reported substantially higher levels of COVID-induced psychoemotional strain.

The article proceeds to theoretical considerations on the relationship between the burdens of COVID and future optimism, age differences and the role of social cohesion as a resilience factor. Following an overview on the data and methods used, we present the empirical evidence and conclude with a discussion of the findings.

## Theoretical framework

### COVID-strain and future optimism

According to Carver et al. (2010), optimism is a generalized positive expectation for one’s future. The key element of optimism lies in the focus on future expectations, intuitively asserting that optimists predominantly expect something positive to happen in the future, whereas pessimists - something negative. Individuals’ optimism is central to how they deal with and cope with adverse events, implying that optimism tends to be a positive response to difficult life trajectories (Nes, 2016).

To begin with, we offer a theoretical mechanism regarding the relationship between COVID-affectedness and optimism for the future. For this purpose, we adapt Veenhoven’s (2012) Sequence Model which was originally formulated with the aim to establish the process behind the formation of individuals’ evaluation of their life-as-a-whole in terms of life satisfaction (happiness). Substituting life evaluation with future optimism, we introduce the model backwards. Optimism for the future (Step 4) is an evaluation based on the flow of life experiences (Step 3) which encompass positive and negative emotions and cognitions. These emotions and cognitions result from various events that people are confronted with in their daily lives (Step 2). The events, though partly random, depend systematically on individuals’ life chances (Step 1). The latter include personal capabilities and resources, but also external societal conditions.

All other life chances being equal, the Corona pandemic—a large-scale external contextual condition—induces many more unpleasant than pleasant situations for the individual, as already discussed earlier in this article. The supposed prevalence of negative situations triggers, in turn, negative emotions and cognitions, the experiences of which lead individuals to regard their future rather with pessimism than with optimism.

### Youth vs. elderly during the COVID-19 pandemic

Despite the overall negative relationship between COVID-induced strain and optimism for the future that logically follows from the Sequence Model, there seems to be room for differences along age. According to the Socioemotional Selectivity Theory (SST; Carstensen, 1991; Carstensen, 1995), the elderly tend to focus on positive experiences rather than worrying about life because they are more aware of the temporal limitedness of life. During the pandemic, on the one side, younger people experienced the lockdown as more difficult (Lehmann et al., 2021), and on the other side, elderly people were at risk of health problems in the case of infection with the virus (Hawkley and Cacioppo, 2007; National Academies of Sciences, Engineering, and Medicine, 2020).

The call for protecting the vulnerable and ‘stepping back’ was mainly aimed at young individuals. Although an infection with the virus would typically progress with mild symptoms for them, youth were expected to keep a low profile, discontinue their usually active lifestyles, quit travelling and abstain from meeting others. As a result, young people stayed home, followed online classes, and postponed their future plans until “after” the pandemic, without knowing when the “after” would come. As a result, almost half of the young students suffered from mental health problems and recovered only slowly after COVID-19 outbreak control, as found to be the case for Chinese students (Gong et al., 2021). Of course, regardless of age, many have suffered from isolation and loneliness, as mentioned earlier. A comparative study of child and adolescent mental health in 59 countries (COH-FIT for short) demonstrated the severity of problems and the need to closely monitor recent trends (Solmi et al., 2022).

The pandemic brought about changes also for people of active age, particularly in terms of work-life balance as a result of disruptions in employment, home-office or child-care arrangements. This resulted in a higher demand for professional psychological support during the lockdown, as shown in a French study of the adult population (Alleaume et al., 2021).

The elderly, on the other hand, the majority of whom were already of retirement age, have usually not experienced the above-mentioned strains, but instead often suffered under social isolation and health concerns (Grolli et al., 2021). It is not exactly clear to what extent the elderly felt discouraged about their future. They have had a higher need of protection against a COVID-19 infection, as the risk of serious infection or death is fundamentally higher than for young people. Yet, the elderly can be assumed to have generally accomplished their personal and professional goals, and to have developed a certain sense of fulfillment. If society sticks together and follows the rules and regulations put up by the authorities, especially the elderly people should rather benefit from the compliance of others. It, thus, appears that youth paid the highest toll for abiding by the rules and regulations during the pandemic.

### Social cohesion as resilience factor

Yet, were all people or all members of an age group similarly affected by the Corona pandemic? Or could some of them rely on a “buffer,” a kind of a protective factor, that makes them resilient against the assumed negative impact of Corona-affectedness on optimism for the future?

In psychology, resilience has generally been described as the capacity to maintain mental health despite adversity (Wald et al., 2006) or, in other words, the “ability to bounce back” (Smith et al., 2008). This refers to humans’ ability to adapt in the face of demanding situations and recover from them. Resilience describes “the capacity of individuals to cope successfully with significant change, adversity or risk” (Lee and Cranford, 2008, p. 213).

Resilience was initially the theoretical basis for defining a personality trait that enables one to withstand adverse situations. Research quickly evolved to define contextual factors closer like negative life experiences, environmental conditions, disadvantages, and risk factors, as well as protective factors for individual development (Werner and Smith, 1977, 1982; Rutter, 1979; Garmezy, 1991). Later, the development of resilience proved to be a process that depended not only on personality, but also on support systems and the broader social environment (Luthar et al., 2000). According to the Bronfenbrenner model, protective factors lie in culture (macrosystem), societal structure (exosystem), the immediate environment, e.g., the neighborhood (mesosystem), the family (microsystem), and the individual (Bronfenbrenner, 1994). More recently, the focus shifted to identifying how protective factors can actively support people in difficult situations (Luthar et al., 2000), rather in a flexible and dynamic process than in a static trait.

Masten (1994) defines three different types of situations in which resilience can play a role. The first situation is when individuals perform better than expected with regard to the adversity they faced. Second, resilience would be said to matter when someone adapts positively throughout the process of coping with an adverse experience. Compared to the first situation, where the focus is on the outcome, the second type emphasizes positive adaptation during the whole event. Finally, individuals can be seen as resilient, if they have suffered severe trauma and have yet recovered from it.

In this study, resilience is understood as a dynamic process that demonstrates positive adaptation to respond to an adverse, stressful situation such as the Corona pandemic. We attempt to understand how individuals who feel the burden of the pandemic assess their future prospects and how this relationship can be mitigated by various possible “protective” social contextual factors such as social networks, diverse environments, or civic engagement. A comprehensive framework accounting for this type of factors is, in our view, the concept of social cohesion.

Social cohesion denotes the “togetherness” of a society to act and appear as one entity and “stick together” in all its heterogeneity (Chan et al., 2006; Dragolov et al., 2016). The concept has gained popularity in both academic research and politics since the end of the Cold War. However, the concept of an inclusive social entity must not be seen as a novelty by itself. Its academic roots can be traced back to Durkheim (1977 [1893]) and Tönnies (1955 [1887]), but undoubtedly one of the slogans of the French Revolution—*fraternité*—should be seen as the onset of public, political, and academic interest in the topic. Possible explanations for the recent rise in attention to social cohesion include widespread concerns that cohesion is being undermined by profound societal changes such as increasing ethnocultural diversity, growing income inequality (Wilkinson and Pickett, 2010), secularization, and technological change (Castells, 1998). Major events from the recent past such as the financial crisis of 2007–2008, the euro crisis of 2008–2011, and more recently the so-called refugee crisis, the Corona pandemic or the military conflict in Ukraine, have reinforced these concerns and the perception of diminishing cohesion in contemporary (post-)modern societies—particularly in the West (Eckersley, 2012; Elchardus and De Keere, 2013).

**Figure 1:**
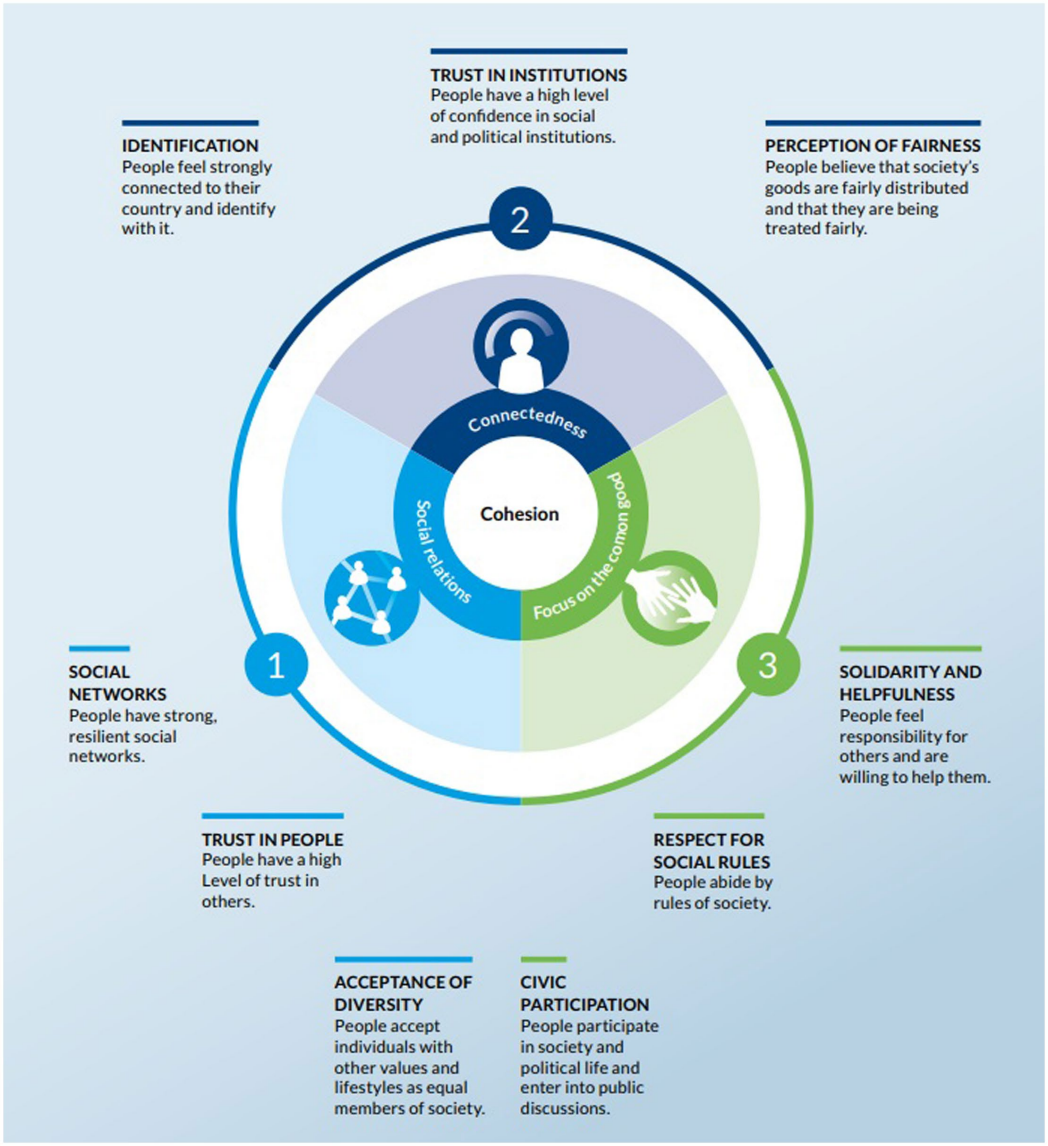
Theoretical framework of the social cohesion radar by Bertelsmann Stiftung.

With its pluralistic, interdependent aspects, the concept of social cohesion is multidimensional. A comprehensive literature review by Schiefer and van der Noll (2017) identifies three theoretically derived domains of social cohesion: social relations, identification with the geopolitical unit, and orientation toward the common good. The authors define social cohesion as a “descriptive attribute of a collective, indicating the quality of collective togetherness” (Schiefer and van der Noll, 2017, p. 592). A cohesive society is characterized by supportive, trust-based, and inclusive social relations; a sense of belonging to the entity, trust in its institutions, and the perception of fairness; solidarity with the weak members, respect for social rules, and active participation in civic life. This definition of social cohesion has been adopted in the *Social Cohesion Radar* of Bertelsmann Stiftung (see Figure 1), which the present study draws on. It has been applied to comparisons of cohesion across countries (see Dragolov et al., 2016, for a comparison of 34 Western societies, Dragolov et al., 2018, for a comparison of 22 Asian societies), regions of Germany (see Arant et al., 2017, for a comparison of 79 regions), and neighborhoods (see Arant et al., 2016, for a comparison of neighborhoods of Bremen). The Baden-Württemberg studies reported here belong to the series looking at subnational units within Germany.

It must be clear that social cohesion is not resilience *per se*. Nevertheless, our aim is to uncover whether it facilitates the resilience of individuals against the strains induced by the Corona pandemic. It seems plausible to assume that the experience of stronger social cohesion reduces the negative impact of the COVID-induced strain on future optimism. Social cohesion is likely to act as a “buffer” or source of protective force that, while not preventing people from being burdened by the pandemic, would at least be in the back of people’s minds when they think about their future. It is unclear, however, whether the buffering effect is age-graded or not. By and large, the elderly can be assumed to benefit from greater social cohesion. They are cared for better and do not have to fear for the—shorter, remaining—future, if social cohesion is high in their residential area. In contrast, it is questionable whether this applies to the case of youth, too. On the one hand, high social cohesion could very well be a general buffer against the severity of COVID-induced strain, but it could also be seen by the young as an additional source of burden due to the high degree of compliance involved.

**Figure 2:**
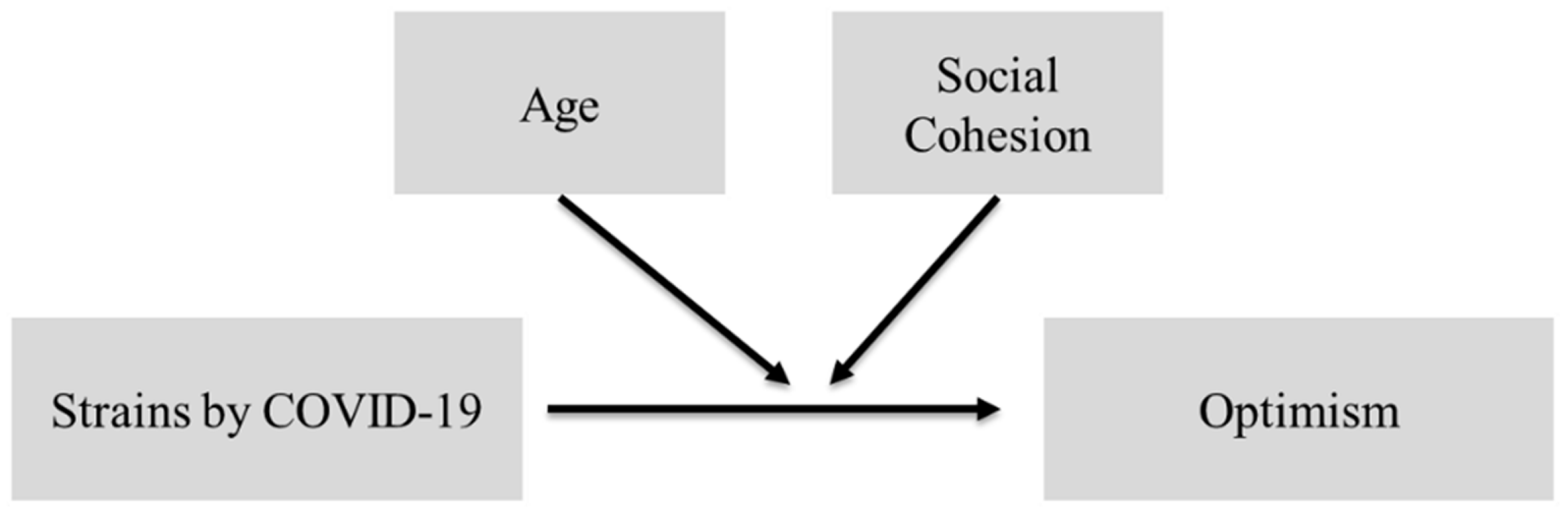
Relationship between COVID-strains and future optimism.

We, thus, expect that perceived social cohesion in people’s residential area buffers the negative repercussions of COVID-strain on future optimism, an effect likely moderated by age, stronger among the elderly, weaker among the young. Figure 2 visualizes our theoretical proposition, treating age and social cohesion as possible moderators between COVID-strain and optimism for the future.

## Data and method

### Data

The data stem from Bertelsmann Stiftung’s *Social Cohesion Radar* (SCR) series on Germany and the federal state of Baden-Württemberg, in particular. The main objective of the SCR is to determine whether and how cohesion on a local and regional level has changed over time. In total, three representative surveys were conducted in 2017, 2019, and 2021/22. In 2017, a Germany-wide CATI^2^ survey of residents aged 16 and above—conducted by the *infas* Institute for Applied Social Science^3^—included a subsample of *N* = 508 individuals from Baden-Württemberg. In 2019 and 2021/2022, representative samples of residents of Baden-Württemberg were surveyed. A CATI survey was conducted in January and February 2019 among *N* = 1,399 individuals by *infratest dimap*,^4^ and an online survey in December 2021 and January 2022 among *N* = 2,716 individuals by *Norstat GmbH*^5^. All three surveys can be considered as representative for the population along major socio-demographic characteristics. For the most recent survey in particular, a quota sampling strategy was used with additional post-hoc calibration of the data based on the official statistics on the population composition with respect to biological sex, age, household income, educational level and household size. The online survey consisted of forced-choice questions, so no data were missing.

### Instruments

All datasets include the SCR questionnaire on individual perceptions of all nine dimensions of social cohesion. English translations of the exact item wordings can be found in Supplementary Table A1 of the Supplementary Appendix. In addition, the 2021/22 survey included several questions on general health, such as a self-assessment of one’s health status and the presence of a chronic illness, on psychological conditions such as the experience of psychological stress and anxiety in relation to the COVID-19 pandemic, and optimism for the future. A special battery of questions was dedicated to respondents’ experience with Sars-CoV-2 such as whether the respondent had direct contact with the virus, or a close person was suffering or had suffered from a COVID-19 infection. In particular, the present article draws on the following instruments.

### Optimism

Respondents’ optimism for the future was assessed with a single item. Respondents were presented with the statement: “I am optimistic about the future,” which had to be rated on a 5-point Likert scale from “strongly disagree” (coded as 1) to “strongly agree” (5). A higher numeric code stands for greater optimism.

### Strain due to the corona pandemic

The measurement of the strain experienced due to the Corona pandemic differentiates between objective and subjective aspects. We operationalize objective strain as the frequency of self-reported health-related experiences with COVID-19. The items to be answered with “yes” or “no” read as follows: “I have tested positive for COVID-19,” “I myself have or have had severe COVID-19 symptoms,” “People close to me have or have had severe COVID-19 symptoms,” and “People close to me have died from an infection with COVID-19.” The resulting index variable ranges from 0 (no experience at all) to 4 (all possible outcomes). Table 1 documents the frequency of the “yes”-answers to the items; Table 2 displays the extent of the experienced objective strain.

**Table 1:** “Yes” responses to aspects of objective strain by COVID-19.

Item	Frequency (%)
I have tested positive for COVID-19.	212 (7.8)
I myself have or have had severe COVID-19 symptoms.	84 (3.1)
People close to me have or have had severe COVID-19 symptoms.	662 (24.4)
People close to me have died from an infection with COVID-19.	228 (8.4)

**Table 2:** Index of objective strain by COVID-19.

Item	Frequency (%)
No objective strain	1880 (69.2)
1 strain	561 (20.7)
2 strains	218 (8.0)
3 strains	39 (1.4)
4 strains	18 (0.7)

Psychoemotional strain, the more subjective aspect, was measured using six items, most of which stem from the COVID-19 Peritraumatic Distress Index (CPDI), originally developed by Qiu et al. (2020). The index addresses topics of psychological distress like social and general anxieties, depression, and panic disorder. Respondents were asked to report how frequently they experienced each of the six issues since the beginning of the Corona pandemic (see Table 3 for the item wording). The response options were “never” (coded as 1), “occasionally” (2), “sometimes” (3), “often” (4), “always” (5). The factor structure was tested in an exploratory factor analysis (EFA), where all six items loaded sufficiently high on the first factor with an eigenvalue of 4.06 (see Table 3). Cronbach’s alpha was calculated to assess the internal consistency of the six-item scale on psychoemotional strain. The internal consistency emerged at α = 0.90. The scale score for psychoemotional strain was computed as the arithmetic mean of the items.

**Table 3:** Exploratory factor analysis of items on psychoemotional strain due to COVID-19.

Item	Factor loading
Compared to usual, I feel more nervous and anxious.	0.74
I am more irritable and have frequent conflicts with my family.	0.71
I feel tired and sometimes even exhausted.	0.83
I find it hard to concentrate.	0.87
I find it hard to make any decisions.	0.84
I feel uncomfortable when communicating with others.	0.71

Unrotated solution. Weighted data from 2021/22 (*N* = 2,716).

### Social cohesion

The degree of social cohesion is assessed along its nine dimensions (see Figure 1 above), resulting in an overall social cohesion index. Since social cohesion is defined as a characteristic of a geopolitical entity, not of single individuals, the individual social cohesion score used in the subsequent analyses should be taken as an indicator for the social cohesion in people’s area of residence as perceived by the individual respondent. Scores for each dimension are calculated as the arithmetic mean of the respective items. The study calculates index scores from indicators for each of the nine dimensions, ranging from 0 (weakest possible degree perceived) to 100 (strongest possible degree perceived). As we use social cohesion as a moderator variable in the subsequent analyses, the sample was split at the score of 50 (the implied mean of the scale) with respect to each dimension and the overall index of social cohesion. In terms of content, the use of the implicit mean appears more meaningful for the interpretation of the results than the empirical mean, median, or standard deviation, which may vary between different target groups. We, thereby, compare two groups of respondents: those who experience a low level of social cohesion (50 or below) and those who experience a high degree (greater than 50).

### Age

The study categorizes individuals into three age groups, following the OECD definition of youth: “those between 15 and 29 years of age” (OECD, 2022). Young respondents are 16 to 29 years old (*n*_1_ = 609), respondents of active age are 30 to 64 years old (*n*_2_ = 1,518) and elderly respondents are of age 65 and above (*n*_3_ = 589).

### Procedure

To set the stage, so-to-speak, the first step was to analyze the general trends in the development of social cohesion in Baden-Württemberg since 2017. For this step, data from 2017, 2019, and 2021/2022 were considered. Sample characteristics of the 2017 and the 2019 studies are documented in Supplementary Tables A2, A3 of the Supplementary Appendix.

In a second step, our analyses utilized the sample from 2021/22, and examined the relationship between objective and psychoemotional COVID-induced strain, on the one hand, and future optimism. Thirdly, analyses (based on the 2021/22 dataset, again) turned to testing for age group differences and the possible moderator role of social cohesion in the relationship between COVID-induced strain and future optimism. These analyses were carried out in a series of multigroup comparisons in the framework of structural equation modeling. All analyses were performed in Stata 17 and apply the calculated data weights.

## Results

### Descriptive results for the 2021/22 study

Participants were on average 47 years old (*SD* = 17.89), with the youngest respondent being 16 and the oldest 96 years of age; 51% of the participants were female. On average, they had a secondary level of education and a relatively high net income compared to the average income in Germany (Statistisches Bundesamt, 2021; see Table 4). Of the participants, 41% reported having no chronic illness.

**Table 4:** Descriptive statistics for the overall sample, three age groups, and experience of social cohesion in 2021/22.

Variable	Overall (*n* = 2,716)		Age groups						Perceived social cohesion			
			Youth (*n* = 609)		Active (*n* = 1,518)		Elderly (*n* = 589)		Low (*n* = 897)		High (*n* = 1819)	
	Mean	*SD*	Mean	*SD*	Mean	*SD*	Mean	*SD*	Mean	*SD*	Mean	*SD*
	Frequency (%)		Frequency (%)		Frequency (%)		Frequency (%)		Frequency (%)		Frequency (%)	
Age	46.63	17.89	22.37	3.71	47.18	10.23	70.29	4.75	46.09	16.72	46.90	18.44
Female gender^a^	1,380 (51)		374 (61)		767 (51)		239 (41)		527 (59)		853 (47)	
Chronic illness^a^^,^^b^	1,125 (41)		166 (27)		636 (42)		323 (559)		430 (48)		695 (38)	
Future optimism	3.18	0.99	3.36	0.97	3.10	1.01	3.20	0.95	2.60	0.99	3.47	0.86
Objective strain	0.44	0.76	0.66	0.91	0.39	0.71	0.33	0.65	0.37	0.73	0.47	0.77
Psychoemotional strain	2.32	0.98	2.84	0.94	2.31	0.96	1.81	0.78	2.61	1.03	2.18	0.93
Social cohesion index	54.32	11.53	54.70	10.01	53.24	11.94	56.69	11.58	41.33	7.15	60.72	7.01

a For dichotomous variables percentages rounded to the full integer are reported. Absolute and relative frequencies are presented.

b Percentages refer to participants responding “yes” to the question whether they suffer from a chronic illness.

The dependent variable used in our study was optimism for the future. Overall, participants indicated only slight optimism for the future (*M* = 3.18, range = 1–5). Optimism is *highest* among young participants (*M* = 3.36), followed by the elderly (*M* = 3.20) and those of active age (*M* = 3.10). As expected, participants experiencing rather low social cohesion report lower optimism for the future (*M* = 2.60) than those who perceive higher cohesion (*M* = 3.47).

Interestingly, a two-thirds majority of the population reported no objective strain due to COVID-19 (score ‘0’). The reported extent of the objective strain was found relatively *highest* among young participants (*M* = 0.66), lower in the group of respondents of active age (*M* = 0.39), and lowest among the elderly (*M* = 0.33). It, thus, appears that young people were, on average, most strongly affected by COVID-19 on objective grounds. Readers should, however, keep in mind that objective strain could take scores between “0” and “4,” meaning that the objective COVID-burden of the studied representative sample was in absolute terms low. Interestingly, incidence rates of objective COVID-burdens were relatively higher among study participants in the high cohesion group (*M* = 0.47) than among those in the low cohesion group (*M* = 0.37).

The extent of psychoemotional strain differs from the population mean (*M* = 2.32) mainly across age groups. The younger the participants, the higher the COVID-induced psychoemotional strain. On average, the active age group (*M* = 2.31) and the elderly (*M* = 1.81) perceive the pandemic as less emotionally burdensome than the youth do (*M* = 2.84). The same is true for participants who perceive a low level of cohesion in their area (*M* = 2.61), meaning that they are more likely to be psychoemotionally affected by the pandemic than those who perceive higher social cohesion (*M* = 2.18). The subjective experience of social cohesion itself also differs across age groups. Older people perceive their area as more cohesive (*M* = 56.69) than, for example, the youth (*M* = 54.70). The descriptive results are shown in Table 4.

### Changes in social cohesion over time by age group

Most studies on social cohesion from before the Corona pandemic demonstrated empirically a high level of stability in social cohesion over time (Dragolov et al., 2016; Delhey et al., 2018). In Baden-Württemberg, overall scores for social cohesion increased minutely from 2017 to 2019 (see Table 5). The slight increase from 63.0 to 63.8 was driven, so-to-speak, by younger participants, whose perception of cohesion increased by nearly 5 points within the two-year period. At the same time, older participants report the highest scores for perceived social cohesion at all three time points. In 2021/22, however, the stability or even upward trend in the experience of social cohesion ceased. Across all three age groups, the overall social cohesion index dropped by about 10 points, relatively least for the elderly.

**Table 5:** Descriptive statistics of social cohesion over time by age groups.

	Social cohesion index					
	2017		2019		2021/22	
	Mean	*SD*	Mean	*SD*	Mean	*SD*
Young (16–29 years)	59.67	9.94	64.56	9.99	54.70	10.02
Active age (30–64 years)	63.52	10.01	63.20	7.71	53.24	11.94
Elderly (65 + years)	65.08	9.15	64.82	9.46	56.69	11.58
Overall	63.00	10.09	63.83	9.67	54.32	11.53

The social cohesion index ranges from 0 to 100.

### Optimism and COVID-19-related strain

Table 6 documents the results from multigroup comparisons, (independently) relating optimism with objective and psychoemotional COVID-induced strain, for the 2021/22 overall sample and separately for the three age groups. Contrary to what one would expect, optimism turns out to be positively related to the objective COVID-induced strain, but effect sizes are minute (ß = 0.08, *p* ≤ 0.01). *People seem to express more optimism, the more they have been objectively affected by COVID-19.*

**Table 6:** Optimism and COVID-19-induced strain.

Predictor variable	Age group	Full sample	Subsamples by age
Objective strain		0.08**	
	16–29		0.06
	30–64		0.07**
	65+		0.02
			
Psychoemotional strain		−0.21**	
	16–29		−0.17**
	30–64		−0.24**
	65+		−0.31**

The dependent variable is optimism. The table documents standardized regression coefficients (*ß*); significance of the estimates is assessed in two-sided tests: **p ≤ 0.01, *p ≤ 0.05, †*p* ≤ 0.10.

Psychoemotional strain is related to future optimism in the to-be-expected way: The stronger the psychoemotional strain participants report, the less optimistic they are for the future (*ß* = −0.21, *p* ≤ 0.01). Age matters for this relationship: It is strongest for the oldest group (*ß* = −0.31, *p* ≤ 0.01) and differs significantly (*p* ≤ 0.05) from the other two age groups.

### Overall social cohesion as a moderator

Table 7 documents the same relationships as analyzed in the above section, now, however, broken down by low vs. high perceived social cohesion. The result is obvious: Neither for objective nor for psychoemotional strain is there a significant difference in effect sizes of the relationship of these variables with future optimism between participants who perceive low vs. high cohesion in their surroundings. There are, however, interesting differences among the three age groups. In the low perceived cohesion condition, no relationship between psychoemotional strain and future optimism emerges for the young, whereas for the two older groups the negative relationship persists. The young differ significantly from the oldest age group (*p* ≤ 0.05) in the low cohesion condition.

**Table 7:** Optimism and COVID-19-induced strain broken down by level of perceived social cohesion.

Predictor variable		Cohesion		
	Age group	Low	High	Δ
Objective strain				
	16–29	−0.01	0.06	0.46 (1)
	30–64	0.04	0.05	0.00 (1)
	65+	−0.04	−0.07	0.00 (1)
				
Psychoemotional Strain				
	16–29	0.02	−0.14*	1.46 (1)
	30–64	−0.17**	−0.17**	0.00 (1)
	65+	−0.32**	−0.23**	0.29 (1)

The dependent variable is optimism. The table documents standardized regression coefficients (*ß*); significance of the estimates is assessed in two-sided tests: **p ≤ 0.01, *p ≤ 0.05, †*p* ≤ 0.10. Column Δ shows the result of a chi-square test of the regression estimates between the low-and high-cohesion groups within the respective age group, where a significant difference is marked with an asterisk.

### Dimensions of social cohesion as moderators

Table 8 gives a comprehensive overview of all standardized regression coefficients for the relationship between objective and psychoemotional COVID-induced strain and future optimism, broken down not only by age group but also by perceived social cohesion (low vs. high), separately for each of its nine dimensions. An inspection of the nine coefficients obtained for the low and the nine coefficients obtained for the high cohesion condition shows that one previously incurred result persists: *When the self-reported objective COVID-induced strain is high, this speaks for more future optimism*. A positive relationship was found for the young (in 15 out of 18 cases, *p* = 0.003)^6^ and the middle-aged group (in all 18 cases, *p* < 0.001), not, however, among the elderly (10 of 18 cases, *p* = 0.167). The opposite emerged for psycho-emotional strain: Only two out of the altogether 54 standardized coefficients have a positive sign, *suggesting that high levels of psychoemotional strain covary strongly with lower optimism*.

**Table 8:** Multigroup comparison of optimism and COVID-induced strain by age group and perceived level of social cohesion.

Dimension of social cohesion	Strength of dimension		
Predictor	Low	High	Δ
*Dimension 1.1 – Social networks*			
Objective strain			
16–29 years	0.21*#	0.00	3.33 (1)†
30–64 years	0.11†	0.02	2.06 (1)
65+ years	−0.04	0.02	0.59 (1)
Psychoemotional strain			
16–29 years	−0.34**	−0.10#	3.04 (1)†
30–64 years	−0.22**	−0.22**	0.01 (1)
65+ years	−0.33**	−0.28**	0.19 (1)
*Dimension 1.2 – Trust in people*			
Objective Strain			
16–29 years	0.05	0.10	0.22 (1)
30–64 years	0.08†	−0.00	2.06 (1)
65+ years	−0.06	0.03	1.09 (1)
Psychoemotional strain			
16–29 years	−0.12†#	−0.19†	0.21 (1)
30–64 years	−0.22**	−0.16**	0.56 (1)
65+ years	−0.31**	−0.27**	0.00 (1)
*Dimension 1.3 – Acceptance of diversity*			
Objective strain			
16–29 years	0.51**#	−0.00	8.07 (1)**
30–64 years	0.02	0.08*	0.66 (1)
65+ years	−0.16†	0.03	3.27 (1)†
Psychoemotional strain			
16–29 years	−0.25	−0.16**	0.18 (1)
30–64 years	−0.22**^	−0.23**	0.09 (1)
65+ years	−0.45**	−0.29**	2.37 (1)
*Dimension 2.1 – Identification*			
Objective strain			
16–29 years	−0.01	0.10	1.10 (1)
30–64 years	0.09*	0.06†	0.44 (1)
65+ years	0.09	−0.02	1.15 (1)
Psychoemotional strain			
16–29 years	−0.11#°	−0.19*	0.52 (1)
30–64 years	−0.32**	−0.17**	5.33 (1)*
65+ years	−0.36**	−0.28**	1.16 (1)
*Dimension 2.2 – Trust in institutions*			
Objective strain			
16–29 years	−0.04	0.23**°	6.04 (1)**
30–64 years	0.04	0.03	0.10 (1)
65+ years	−0.02	0.12†	2.37 (1)
Psychoemotional strain			
16–29 years	−0.11#	−0.16†	0.04 (1)
30–64 years	−0.17**^	−0.20**	0.04 (1)
65+ years	−0.33**	−0.23*	1.44 (1)
*Dimension 2.3 – Perception of fairness*			
Objective strain			
16–29 years	0.05	0.01	0.17 (1)
30–64 years	0.07*	0.03	0.42 (1)
65+ years	0.05	−0.18†	4.96 (1)*
Psychoemotional strain			
16–29 years	−0.23**	0.04	4.37 (1)*
30–64 years	−0.28**	−0.03	7.51 (1)**
65+ years	−0.30**	−0.24†	0.00 (1)
*Dimension 3.1 – Solidarity and helpfulness*			
Objective strain			
16–29 years	0.03	0.09	0.33 (1)
30–64 years	0.06	0.05	0.13 (1)
65+ years	0.05	−0.08	0.22 (1)
Psychoemotional strain			
16–29 years	−0.16*	−0.17#	0.01 (1)
30–64 years	−0.23**	−0.26**^	0.01 (1)
65+ years	−0.29**	−0.37**	1.05 (1)
*Dimension 3.2 – Respect for social rules*			
Objective Strain			
16–29 years	0.06	0.04	0.01 (1)
30–64 years	0.12**	0.07*	0.21 (1)
65+ years	0.10	−0.02	1.46 (1)
Psychoemotional strain			
16–29 years	0.05#	−0.21**	2.36 (1)
30–64 years	−0.15*^	−0.22**	0.79 (1)
65+ years	−0.36**	−0.24**	0.78 (1)
*Dimension 3.3 – Civic Participation*			
Objective Strain			
16–29 years	0.02	0.15	1.08 (1)
30–64 years	0.05†	0.07	0.07 (1)
65+ years	0.04	−0.02	0.59 (1)
Psychoemotional strain			
16–29 years	−0.17**	−0.21	0.07 (1)
30–64 years	−0.19**	−0.33**	4.38 (1)*
65+ years	−0.31**	−0.32**	0.02 (1)

The table documents standardized regression coefficients (*ß*). Significance of the estimates is assessed in two-sided tests: **p ≤ 0.01, *p ≤ 0.05, †*p* ≤ 0.10; # designates a significant difference (*p* ≤ 0.05) between the young and the elderly; ° designates a significant difference (*p* ≤ 0.05) between the young and active age group; ^ designates a significant difference between the active age group and the elderly. Column Δ shows the result of a chi-square test of the regression estimates between the low-and high-cohesion groups within the respective age group, where a significant difference is marked with an asterisk.

The picture is much less clear-cut regarding the question of whether the relationship between objective/psychoemotional strain and future optimism is moderated by the single dimensions of social cohesion. In 13 out of altogether 54 possible instances, we find a significant moderation indicated by a significant difference between the coefficients reported for the high and the low cohesion group. Seven of the 13 cases pertain to objective strain, the other six to psychoemotional strain. In nine of 13 cases effect sizes are larger in the low cohesion group than in the high cohesion group (*p* = 0.087).

## Discussion

The current paper has ventured into disentangling the relationship of optimism for the future, strain arising from COVID-19, and the role of social cohesion in moderating this relationship toward a resilient response of the individual. Data originate from representative survey studies conducted in the German federal state of Baden-Württemberg. The exploratory research endeavor had its starting point in the finding that in contrast to essentially all available prior findings, social cohesion plunged over COVID in the relatively prosperous Baden-Württemberg by about 10 points from 64 down to 54 on a scale of 100: COVID does seem to affect social cohesion strongly negatively. It endangers societal “togetherness,” a societal feature that has usually been quite stable over time.

Findings on the detrimental effects of serious worries about COVID among citizens of Baden-Württemberg were clear-cut: Psychoemotional strain experienced during COVID dampened people’s optimism for the future; yet, more so for the elderly than for youth.

A surprising result emerged for the relationship between objective COVID strain and optimism for the future. Even though effect sizes are small, the finding prevailed that there is some kind of bounce-back effect. It is indeed the case that those study participants who have experienced more objective strain, look more positively into the future. The folklore wisdom that what does not kill us only makes us stronger, seems to be empirically supported. Overcoming COVID appears to be a fountain of optimism for the future.

But how does social cohesion affect the relationship between objective/psychoemotional strain and optimism for the future? Is a high level of social cohesion in one’s residential geopolitical unit indeed a protective factor against the woes and worries of COVID? Do high levels of social cohesion foster a resilience response?

The empirical picture is rather blurry. There is little evidence that the overall level of social cohesion moderates the relationship between strain—objective or psychoemotional—and optimism for the future. Only when one looks at the single dimensions of the formative cohesion index and the different age groups separately, does reasonably strong evidence emerge that social cohesion indeed moderates the relationship between strain and optimism.

For objective strain the bounce-back effect mentioned above was most tangibly moderated by the cohesion level for the dimensions Social Networks (1.1), Acceptance of Diversity (1.3), and Trust in Institutions (2.2) among youth. However, only for Trust in Institutions did the moderation effect have the “right” direction: The bounce-back effect (“what does not kill us only makes us stronger”) was bigger among youth perceiving high cohesion, in this case, high levels of Trust in Institutions (for concrete items see Supplementary Appendix, Supplementary Table A1). In other words, young people who suffer the consequences of Corona and indicate that they trust institutions such as political parties, the police, or parliament seem to be more optimistic about the future than those who have low trust in public institutions. It seems plausible that despite the stress of being ill and/or having close relatives who are ill, it should be relieving to have an institution that ‘has your back when you are unwell.’ Political parties have been one of the driving forces during the pandemic to get quick help on the way. It is only logical that people who have confidence in these parties and in parliament, in general, will also be more optimistic about the future during the crisis.

In the other two instances findings were reversed. Young people who lived in a more aversive (i.e., low cohesion) context regarding social networks and with regard to acceptance of diversity showed a stronger bounce-back effect: When the youth have non-intact social relations and difficulties with accepting diversity in their surroundings, they are *more optimisti*c about their future in light of objective COVID-strain. In terms of social networks, one possible explanation could be that those who have more social connections have ‘more to lose’ than those who have fewer social connections and are therefore more likely to be pessimistic about the future.

Second, this finding emerged as being rather strong for the diversity acceptance dimension, meaning that participants who indicated a more exclusive worldview with an increasing rejection of migrants, people with different lifestyles, or religions were less concerned about the future. One possible explanation for this could be that people with a similar mindset tend to be more egocentrically oriented. The central interest is rather in their immediate environment, without caring so much about “the others who are in any way different from me.” It could be that this is associated with immediate stress reduction. The division into “us” and “them” may make it easier for people to separate themselves emotionally as well. Young people, who have a more inclusive attitude in the first place, may find it burdensome to think about possible disadvantaged groups during the pandemic, which could translate into a more pessimistic attitude toward the future. Should this be a call to be alone and intolerant? Rather not, but a call to action, which we will discuss further below.

For psychoemotional strain, results were slightly more straightforward. Youth in the high cohesion group with regard to the intactness of social networks were indeed less affected in their optimism by psychoemotional strain than were youth in the low cohesion group for this dimension. The same was the case regarding the Perception of Fairness (Dimension 2.3): Youth in the high cohesion group were less affected in their optimism by psychoemotional strain than were youth in the low cohesion group.

In summary, one must conclude that high social cohesion only has very modest safeguarding or protective effect against detrimental consequences of COVID-induced strain for young people’s future optimism. What is more interesting *per se* is that there is a weak but persistent bounce-back effect. Youth, who have had experience with COVID, be it themselves or in their immediate life context, seem to be more resilient in the sense that they preserve their future optimism more than youth who did not have this experience. Psychoemotional strain, i.e., being excessively worried about COVID, seems to be the greater danger for future optimism, but more so for the elderly than for youth.

It is difficult to discuss the results of the current study considering the available literature. Of course, there are studies on psychological repercussion of COVID in abundance. Searches in pertinent data bases (like PsycInfo) return more than 20,000 entries. Studies that work with representative random probability samples and concentrate on comparing youth with other age groups are extremely scarce. Research like the French study by Alleaume et al. (2021) or the Chinese study by Gong et al. (2021) and the so-called COH-FIT-C&A study (Solmi et al., 2022) that provides data from 59 countries with representative samples from 11 countries is a rare exception. We refrain here from contrasting our work with these studies but point to the two obvious limitations of the current research: Its generalizability is limited. Studying the population of a rather prosperous German state is likely to misrepresent youth’s reactions to COVID, when seen from a global perspective. Secondly, its cross-sectional design does not allow even quasi-causal interpretations: We cannot say whether the effects we found are age, cohort, or period effects.

Whether or not the COVID crisis has really been overcome yet, young people have a lot to catch up with in the wake of the crisis—not only in the areas of school, training, and university, but also in terms of leisure time, gaining experience and developing their personalities. A “catch-up package” seems to be the call of the day. Such an offer helps counteract the psychoemotional stresses of young people during the pandemic period. To this end, the relevant institutions—schools, colleges, youth centers—must be provided with the necessary resources. The novel finding reported here is that such ‘packages’ should distinguish between youth who have had immediate personal experience with COVID and youth who have not: To strengthen the bounce-back effect would then be the aim of “packages” for youth who have had personal experience with COVID, whereas reducing feelings of psychoemotional stress should be in focus for those who have not.

## Data availability statement

The raw data supporting the conclusions of this article will be made available by the authors, without undue reservation.

## Ethics statement

Ethical review and approval was not required for the study on human participants in accordance with the local legislation and institutional requirements. Written informed consent from the participants’ legal guardian/next of kin was not required to participate in this study in accordance with the national legislation and the institutional requirements.

## Author contributions

CH conducted analyses, provided a first full draft of the paper, and also the next-to-final draft. GD participated in designing all three survey studies, conducted analyses, and participated in writing the first draft and editing the final draft of the paper. RA was in charge of designing all three studies and provided important comments on the first draft of the paper. JD was co-responsible for developing the conceptual and the measuring approach of the Social Cohesion Radar and offered valuable comments on the next-to-final version of the paper. KU was co-responsible for developing the conceptual and the measuring approach of the Social Cohesion Radar, he facilitated funding of all three reported studies and offered valuable comments on the first draft of the paper. KB was principle investigator of all three reported studies, he also took over a major role in structuring and editing the paper across the entire writing process. All authors contributed to the article and approved the submitted version.

## Funding

All three reported studies were directly or indirectly funded by Bertelsmann Stiftung, Gütersloh, Germany (no formal award number). Studies conducted in 2019 and in 2021/22 were commissioned by the Baden-Württemberg Ministry for Social Affairs, Health, and Integration, who provided funds to Bertelsmann Stiftung to have the studies academically supervised at Constructor University, Bremen by the team of the last author and his collaborators. We are grateful to the Deutsche Forschungsgemeinschaft (DFG, German Research Foundation – GRK 2513 /404484063) for supporting us with the additional funding for publication.

## Conflict of interest

The authors declare that the research was conducted in the absence of any commercial or financial relationships that could be construed as a potential conflict of interest.

## Publisher’s note

All claims expressed in this article are solely those of the authors and do not necessarily represent those of their affiliated organizations, or those of the publisher, the editors and the reviewers. Any product that may be evaluated in this article, or claim that may be made by its manufacturer, is not guaranteed or endorsed by the publisher.

## Footnotes

^1^ https://covid19.who.int

^2^**C**omputer-**A**ssisted **T**elephone **I**nterview.

^3^ https://www.infas.eu

^4^ https://www.infratest-dimap.de

^5^ https://norstat.de/

^6^Significance tested *via* the Binomial Test.
