# Supplementary material for: Youth and social cohesion in times of the COVID pandemic: Most negatively affected? Most resilient?
Source: Front Psychol. 2023 Feb 20;14:1036516. doi: 10.3389/fpsyg.2023.1036516 (PMC10038128; doi:10.3389/fpsyg.2023.1036516)
Supplement: Supplementary file 1 [file Data_Sheet_1.docx]

# Appendix

**Table A1**

*List of Indicators for the Dimensions of the Social Cohesion Index*

| *Dimension 1.1 – Social Networks* | |
| --- | --- |
| How large is your circle of friends and acquaintances?  [very large – rather large – medium – rather small – very small] | |
| How often do you meet socially with friends, relatives or colleagues?  [very often – rather often – sometimes – rather seldom – very seldom] | |
| If you were having difficulties: Do you have friends whose help you can count on at any time? [yes / no] | |
| Do you have friends or acquaintances outside your family who would lend you 1,000 euro in an emergency? [yes / no] | |
| *Dimension 1.2 – Trust in People* | |
| Most people can be trusted. [one can’t be too careful – most people can be trusted] | |
| How much do you trust people you meet for the first time?  [completely – somewhat – neither… nor – not very much – not at all] | |
| I am convinced that most people have good intentions.  [completely true – somewhat true – neither… nor – rather not true – not true at all] | |
| Nowadays, you can't rely on anyone.  [completely true – somewhat true – neither… nor – rather not true – not true at all] | |
| *Dimension 1.3 – Acceptance of Diversity* | |
| Which of the following groups would you not like to have as neighbors: People with a completely different lifestyle? | |
| Which of the following groups would you not like to have as neighbors: people of other religions? | |
| Which of the following groups would you not like to have as neighbors: foreigners / migrants? | |
| Which of the following groups would you not like to have as neighbors: homosexuals? [Yes, would not like to have as neighbors / No, I would not mind] | |
| Would you say that, in general, life in Germany is threatened or enriched by increasing diversity? [threatened – enriched] | |
| *Dimension 2.1 – Identification* | |
| How emotionally attached do you feel to your federal state? | |
| How emotionally attached do you feel to your region? | |
| How emotionally attached do you feel to your place of residence? | |
| How emotionally attached do you feel to your neighborhood?  [very much – somewhat – neither… nor – not much – not at all] | |
| *Dimension 2.2 – Trust in Institutions* | |
| How much do you trust the political parties? | |
| How much do you trust the courts? | |
| How much do you trust the police? | |
| How much do you trust the federal government? | |
| How much do you trust the federal parliament?  [completely – somewhat – neither… nor – not very much – not at all] | |
| *Dimension 2.3 – Perception of Fairness* | |
| Social differences in our country are by and large just. | |
| Differences in rank between people are acceptable because they essentially express, what one has made of the chances one has had. | |
| Economic gains are by and large fairly distributed in Germany today. | |
| In Germany, one is compensated according to their performance.  [completely true – somewhat true – neither… nor – rather not true – not true at all] | |
| *Dimension 3.1 – Solidarity and Helpfulness* | |
| Some people donate money from time to time to social or charitable causes. Have you made such donations in the last 12 months? [yes / no] | |
| How often do you volunteer in your free time in order to help other people, e.g. in associations, organizations, unions or social services?  [every day / at least once a week / at least once a month / less frequently / never] | |
| In reality, most people don't care at all about what happens to their fellow citizens. [completely true – somewhat true – neither… nor – rather not true – not true at all] | |
| *Dimension 3.2 – Respect for Social Rules* | |
| In general, how do you feel in your neighborhood?  [very safe – rather safe – neither… nor – rather unsafe – very unsafe] | |
| Is there actually any area around here, where you wouldn’t want to go alone at night? [yes / no] | |
| Are there any problems in your neighborhood with: dog dirt? [yes / no]  Are there any problems in your neighborhood with: wrongly parked cars? [yes / no]  Are there any problems in your neighborhood with: damaged playgrounds? [yes / no]  Are there any problems in your neighborhood with: graffiti ? [yes / no]  Are there any problems in your neighborhood with: garbage on streets? [yes / no]  Are there any problems in your neighborhood with: disorderly conduct? [yes / no]  Are there any problems in your neighborhood with: drunkards? [yes / no]  Are there any problems in your neighborhood with: harassment? [yes / no] | |
| *Dimension 3.3 – Civic Participation* | |
| In the past 12 months, have you: participated in a demonstration? [yes / no]  In the past 12 months, have you: participated in a petition? [yes / no]  In the past 12 months, have you: displayed a badge or a sticker? [yes / no]  In the past 12 months, have you: had a political position? [yes / no]  In the past 12 months, have you: participated in a local initiative? [yes / no]  In the past 12 months, have you: participated in a town hall meeting? [yes / no]  In the past 12 months, have you: contacted a politician? [yes / no]  In the past 12 months, have you: taken a political stance on social media? [yes / no] | |
| How strong is your interest in politics?  [very strong – strong – medium – little – not at all] | |
| If the next elections, for which you are eligible, are coming up, how likely are you to actually vote? [very likely – rather likely – rather unlikely – very unlikely] | |
| Are you a member of a nonprofit association or organization? [yes / no] | |

**Table A2**

*Descriptive Statistics for the Overall Sample, by Age Group and Perceived Social Cohesion in 2017*

|  | Overall  (*n* = 646) | | Age Groups | | | | | | Perceived Social Cohesion | | | |
| --- | --- | --- | --- | --- | --- | --- | --- | --- | --- | --- | --- | --- |
|  |  |  | Youth  (*n* = 120) | | Active  (*n* = 368) | | Elderly  (*n* = 139) | | Low  (*n* = 66) | | High  (*n* = 580) | |
|  | Mean | *SD* | Mean | *SD* | Mean | *SD* | Mean | *SD* | Mean | *SD* | Mean | *SD* |
| Variable | Frequency (%) | | Frequency (%) | | Frequency (%) | | Frequency (%) | | Frequency (%) | | Frequency (%) | |
| Age | 48.21 | 18.39 | 22.80 | 4.21 | 47.03 | 10.38 | 73.23 | 5.88 | 44.06 | 18.72 | 48.67 | 18.32 |
| Female gender^a^ | 339 (53) | | 58 (48) | | 190 (52) | | 82 (59) | | 36 (54) | | 304 (52) | |
| Future optimism | 3.48 | 1.01 | 3.50 | 1.18 | 3.57 | 0.98 | 3.32 | 0.87 | 2.67 | 1.30 | 3.58 | 0.93 |
| Social Cohesion Index | 63.00 | 10.09 | 59.67 | 10.01 | 63.52 | 10.20 | 65.08 | 9.15 | 43.15 | 5.16 | 65.25 | 7.78 |

*Note*. Unlike the data in 2019 and 2021/22, there was no information on the chronic illnesses of the participants in 2017.

^a^ For dichotomous variables percentages rounded to the full integer are reported. Absolute and relative frequencies are presented.

**Table A3**

*Descriptive Statistics for the Overall Sample, by Age Groups and Perceived Social Cohesion in 2019*

|  | Overall  (*n* = 1399) | | Age Groups | | | | | | Perceived Social Cohesion | | | |
| --- | --- | --- | --- | --- | --- | --- | --- | --- | --- | --- | --- | --- |
|  |  |  | Youth  (*n* = 211) | | Active  (*n* = 823) | | Elderly  (*n* = 360) | | Low  (*n* = 127) | | High  (*n* = 1272) | |
|  | Mean | *SD* | Mean | *SD* | Mean | *SD* | Mean | *SD* | Mean | *SD* | Mean | *SD* |
| Variable | Frequency (%) | | Frequency (%) | | Frequency (%) | | Frequency (%) | | Frequency (%) | | Frequency (%) | |
| Age | 50.15 | 18.79 | 21.35 | 4.23 | 47.24 | 10.52 | 73.69 | 6.53 | 48.84 | 17.42 | 50.28 | 18.92 |
| Female gender^a^ | 714 (51) | | 100 (47) | | 413 (50) | | 203 (57) | | 73 (58) | | 645 (51) | |
| Chronic illnesses^a^ | 448 (32) | | 32 (15) | | 269 (33) | | 147 (41) | | 71 (56) | | 377 (30) | |
| Future optimism | 3.73 | 1.00 | 3.98 | 1.01 | 3.71 | 1.01 | 3.62 | 0.97 | 2.72 | 1.12 | 3.83 | 0.93 |
| Social Cohesion Index | 63.83 | 9.67 | 64.56 | 7.71 | 63.20 | 10.18 | 64.82 | 9.46 | 43.39 | 5.57 | 65.87 | 7.33 |

*Note*. ^a^ For dichotomous variables percentages rounded to the full integer are reported. Absolute and relative frequencies are presented.
